# Functional characterisation of a novel class of *in-frame* insertion variants of *KRAS* and *HRAS*

**DOI:** 10.1038/s41598-019-44584-7

**Published:** 2019-06-03

**Authors:** Astrid Eijkelenboom, Frederik M. A. van Schaik, Robert M. van Es, Roel W. Ten Broek, Tuula Rinne, Carine van der Vleuten, Uta Flucke, Marjolijn J. L. Ligtenberg, Holger Rehmann

**Affiliations:** 1 Department of Pathology, Radboud university medical center, Nijmegen, The Netherlands; 2 Department of Molecular Cancer Research, Center for Molecular Medicine, Oncode Institute, University Medical Center Utrecht, Utrecht, 3584 CX The Netherlands; 3 Department of Human Genetics, Radboud university medical center, Nijmegen, The Netherlands; 4 Department of Dermatology, Radboudumc Center of Expertise Hecovan, Radboud university medical center, Nijmegen, The Netherlands; 5 Expertise Centre for Structural Biology, University Medical Center Utrecht, Utrecht University, Utrecht, 3584 CX The Netherlands

**Keywords:** Molecular medicine, GTP-binding protein regulators, Oncogenes

## Abstract

Mutations in the *RAS* genes are identified in a variety of clinical settings, ranging from somatic mutations in oncology to germline mutations in developmental disorders, also known as ‘RASopathies’, and vascular malformations/overgrowth syndromes. Generally single amino acid substitutions are identified, that result in an increase of the GTP bound fraction of the RAS proteins causing constitutive signalling. Here, a series of 7 *in-frame* insertions and duplications in *HRAS* (n = 5) and *KRAS* (n = 2) is presented, resulting in the insertion of 7–10 amino acids residues in the switch II region. These variants were identified in routine diagnostic screening of 299 samples for somatic mutations in vascular malformations/overgrowth syndromes (n = 6) and in germline analyses for RASopathies (n = 1). Biophysical characterization shows the inability of Guanine Nucleotide Exchange Factors to induce GTP loading and reduced intrinsic and GAP-stimulated GTP hydrolysis. As a consequence of these opposing effects, increased RAS signalling is detected in a cellular model system. Therefore these in-frame insertions represent a new class of weakly activating clinically relevant *RAS* variants.

## Introduction

Overgrowth syndromes, including vascular malformations represent a spectrum of conditions with congenital, aberrant vascular structures combined with overgrowth of surrounding tissue^1–4^. The identification and classification of vascular malformations/overgrowth syndromes, hereafter referred to as VMOS, can be challenging for physicians, pathologists and clinical geneticists. Usually the combination of clinical course and radiological investigations leads to a diagnosis as defined within the ISSVA classification^1^. VMOS do not display deviant vascular histology in all cases. Histopathological investigation may be indicated to rule out a (vascular) tumour and in particular malignancy.

Vascular malformations may consist of either aberrant capillary, lymphatic, venous or arteriovenous vessels or a combination of those. Depending on the vessel-type, the patient may experience swelling, congestion, disfigurement, (disabling) pain, thrombosis, embolism or bleeding. Although vascular malformations are usually benign conditions, all symptoms mentioned may result in significant complications and morbidity.

This spectrum of lesions is caused by somatic *de novo* mutations that occur during embryonal development resulting in mosaicism or rarely by germline mutations. Depending on the event and location of the mutation, the clinical presentation caused by somatic mutations can resemble those of patients affected by germline mutations. Generally, activating mutations are identified in genes associated with cell proliferation, cell growth, cell cycle regulation, and survival, while inactivating mutations are identified in genes with opposite biological functions^5–8^. Genetic tools for vascular anomalies have broadened the diagnostic scope. Sensitive Next Generation Sequencing (NGS) based approaches can be used to screen for mosaicism, which aids in diagnosis, exclusion of germline causes and allows identification of potentially clinically targetable disruptions^9^.

Germline and somatic mutations in genes coding for proteins of the RAS/MAPK pathway are known to cause a spectrum of congenital diseases named ‘RASopathies’ including Neurofibromatosis type I, Noonan syndrome, and capillary malformation-arteriovenous malformation syndrome^10^. In a subset of cases of VMOS, somatic pathogenic mutations in genes associated with RAS signalling are identified, including mutations that causes enhanced signalling in the *RAS* genes themselves^9,11,12^. RAS mediated signalling is well studied, as somatic mutations in *KRAS* are found commonly in tumours whereas mutations in *HRAS* and *NRAS* are found with much lower frequency. Oncogenic variants are typically found at hotspots in the *RAS* genes causing substitutions of residues Gly12, Gly13, Gln61, Lys117 or Ala146 and are referred to here as ‘classical mutations’.

HRAS, KRAS and NRAS are small G-proteins with high sequence homology. Small G-proteins cycle between a GDP and a GTP bound state, whereby the GTP bound state causes downstream signalling^13,14^. Under physiological conditions, the net transition to the GTP bound state occurs by nucleotide exchange as a dissociated nucleotide is replaced with higher probability by GTP than GDP due to the high cellular concentration of GTP. Nucleotide release is accelerated by Guanine Nucleotide Exchange Factors (GEFs). GEFs induce RAS signalling. Hydrolysis of GTP to GDP and phosphate by the G-protein results in the transition to the GDP bound state. The low intrinsic GTPase activity of small G-proteins is accelerated by several orders of magnitude by GTPase Activating Proteins (GAPs). Thereby GAPs terminate RAS signalling. The conformation of the G-protein, in particular of the so-called switch I and switch II region, depends on the nature of the bound nucleotide. Effector proteins interact selectively with the GTP-bound conformation. An increase of the GTP bound fraction of the G-protein thus enhances downstream RAS signalling. Mutations causing such a shift are therefore referred to as activating.

The molecular effects that result in an increase of the GTP bound fraction differ. Gly12 is localised in the P-loop that is involved in binding of the phosphate moiety of the nucleotide^15^. Missense mutations of Gly12 reduce the rate of intrinsic GTP hydrolysis and the catalytic effect of GAPs. To catalyse GTP hydrolysis, GAPs for RAS proteins provide an arginine residue, the positive charge of which compensates for the negative charge that accumulates at the γ-phosphate during hydrolysis upon the nucleophilic attack by a water molecule. Missense mutations of Gly12 sterically prevent the proper positioning of this arginine residue^16^. The same effect occurs if Gly13 is mutated. The side chain of Gln61 positions the attacking water molecule by a direct hydrogen bond and is itself stabilised in a catalytic competent conformation by RasGAP^15–17^. Hydrolysis is less efficient without proper positioning of the attacking water and in addition mutations of Gln61 reduce the affinity to RasGAP. Lys117 is part of the NKxD motif and Ala146 of the SAK motif. Both motifs are involved in binding the base of the nucleotide^17^. Alterations in this motif result in decreased nucleotide affinity and thus increase nucleotide dissociation rates. As a consequence, intrinsic nucleotide exchange is increased and thereby the fraction of GTP bound RAS.

Here, a series of 7 novel in-frame insertions in *HRAS* and *KRAS*, identified in routine diagnostic screening for somatic mutations in VMOS and germline screening for RASopathies, are functionally characterised. These variants display unique properties and act as weakly but constitutively activating.

## Results

### Patient cohort

NGS based analysis was performed routinely in cases where VMOS were diagnosed based on clinical, radiological, and histopathological evaluation. These were cases in which initially no clear diagnosis of VMOS was possible or where treatment was insufficient with progression of the lesion. In this respect the cases were classified as “atypical” VMOS. In cases of smaller lesions surgical excision and otherwise incision biopsy was performed. Histopathological investigation could exclude malignancy.

### Identification of RAS in-frame insertions

**Figure 1:**
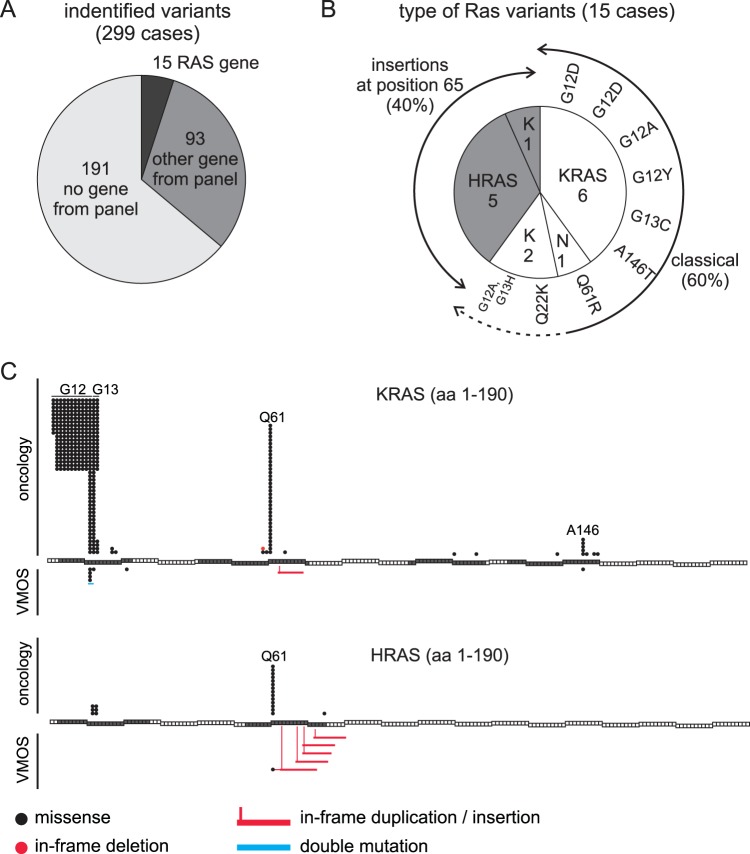
Identification of *RAS* in-frame insertion variants. (**A**) Pie-chart showing potentially causative variants found in 299 diagnostic cases with VMOS. *RAS*: *HRAS*, *KRAS* or *NRAS*; others genes from panel, variation in one of the sequenced genes with exception of *RAS* genes; no gene from panel, no variation in any sequenced gene (details in Materials and Methods). (**B**) Pie-chart of the type of *RAS* variants found. White, classical mutations; grey *in-frame* insertions around amino acid position 65. K, *KRAS*; N, *NRAS*. Mutation on amino acid level. (**C**) Comparison of the *RAS* variants found in cases of VMOS with variants identified in 3234 oncology related diagnostic requests. Each box represents one codon. Grey boxes, codons covered by NGS analysis. The variants found in oncology related diagnostics and VMOS cases are indicated above and underneath of the affected codons, respectively. Each circle/item represents one case.

**Figure 2:**
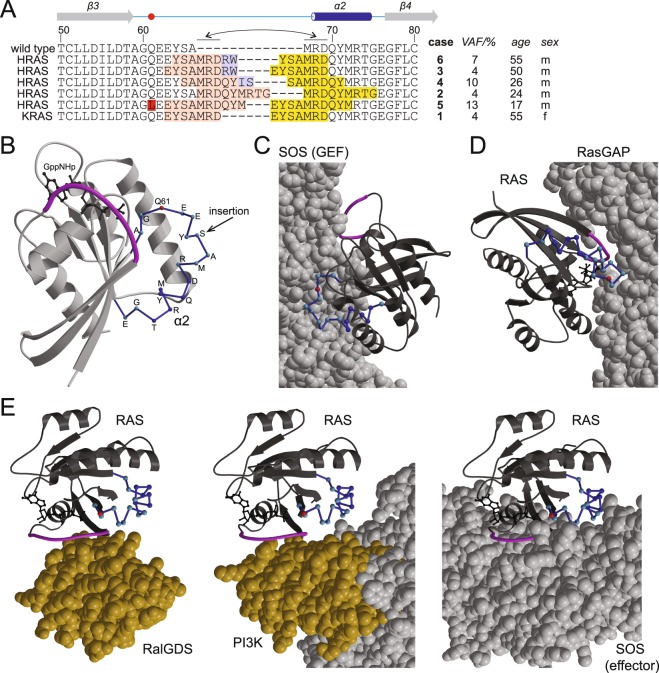
Structural context of VMOS RAS insertions. (**A**) Sequence alignment on amino acid level from position 50 to 80. The sequences of HRAS and KRAS are identical in the aligned region and labelled as wild type. Duplicated sequence is highlighted once by light red and once by yellow background; novel sequence by light blue and point mutations by red background. *Note*: This colour coding is irrespective of the genetic rearrangements on DNA level purely based on the amino acid sequence. Elements of secondary structure are displayed above the alignment, with helix α2 in dark blue and N- and C-terminal connecting loops in light blue. The position of Q61 is marked by a red circle. The affected genes are indicated left to the alignment. Case number, Variant Allele Frequency (VAF), and age and sex of the patient are listed right to the alignment. (**B**) Structure of HRAS in complex with the hydrolysis resistant GTP analogue GppNHp in ribbon representation but with helix α2 and its connecting loops as dark blue backbone trace. Cα-atoms in the backbone trace are shown as colour coded spheres: helix α2, dark blue; connecting loops, light blue; Gln61, red. This region corresponds to switch II. Switch I is highlighted in magenta. Style of representation and colour code is maintained throughout this figure. The likely place of insertion is indicate by an arrow. Dark grey, GppNHp in ball-and-stick representation. (**C**–**E**) Structures of HRAS (ribbon representation, dark grey) in complex with SOS acting as GEF (**C**), RasGAP (**D**), and the effectors RalGDS (E, left), PI3K (E, middle) and SOS (E, right) (space filling representation). Nucleotides are shown in ball-and-stick representation in black: GDP•AlF_3_ (**D**) and GppNHp (**E**). The ubiquitin folds of RalGDS and PI3K mediating the interaction with RAS are coloured in orange. See Supplementary Fig. 1B for RAS in complex with the ubiquitin folds of the effectors RAF^38^, PLCε^39^, and Nore1^40^. *Note:* The orientation of RAS is different in each panel, but identical for all complexes in (**E**) to allow for a free view of the interaction surface. See Supplementary Fig. 1A for a set of figures in identical orientation. Figures were generated base on the pdb database entries 5p21^15^, 1dbk^32^, 1wq1^16^, 1lfd^41^, 1he8^42^, and 1nvv^22^ by use of the programs molscript^43^ and raster3D^44^.

Sensitive NGS based screening of frequently mutated positions in a panel of multiple genes were applied in 299 cases. In 108 cases, putative causative variants were identified, of which in 15 cases *RAS* genes were affected (Fig. 1A). 7 of the 15 variants of *RAS* genes were classical oncogenic mutations in *KRAS* and *NRAS* affecting codons 12, 13, 61 or 146 (Fig. 1B,C). In two cases semi-classical mutations were identified. In one case p.Q22K was observed in *KRAS*. This mutation is rarely found in tumour samples and shown to display increased GTP loading^18^. Another case harboured an in frame deletion-insertion, simultaneously affecting codons 12 and 13 of *KRAS*, resulting in p.G12A and p.G13H. This specific variant was, to the best of our knowledge, not described previously. However, both mutations effect classical positions in the P-loop. It is likely that this variant display increased GTP loading. The remaining 6 cases contained an *in-frame* insertion in *HRAS* or *KRAS* resulting in the insertion of 7 to 10 residues around position 65 (Figs 1B,C and 2A, Supplementary Information). These cases represented 2.0% of all cases and 40% of cases with *RAS* variants (Fig. 1A,B).

Sequence analysis of *HRAS, KRAS* and *NRAS* with an identical approach in 3234 oncology diagnostic requests, resulted in the identification of 354 variants of *KRAS* and 21 variants of *HRAS*. The observed mutational spectrum is in line with the literature^19^ and did not reveal any insertion or duplication variants around position 65 (Fig. 1C). This suggests that the *RAS in-frame* insertion variants are disease-specific. Therefore, the 6 *in-frame* insertion variants detected in VMOS will be referred to as VMOS *RAS* variants.

### Clinical Characteristics of VMOS RAS variants

All patients with *in-frame* insertion variants of *KRAS* and *HRAS* were (young) adults (Supplementary Information). All patients except one were male and had relatively large (5 to 10 cm) progressive and usually painful swellings of arm, leg, abdominal wall, back musculature or face. Clinically, the swellings appeared as vascular or lipoma-like. Radiological investigation by ultrasound or MRI showed vascular characteristics, sometimes with high flow, but without the characteristics of arteriovenous malformation (AVM) at angiography (case 3).

### *In silico* analysis of VMOS RAS variants

On amino acid level the DNA duplications and insertions found in the VMOS RAS variants resulted in an insertion of mainly duplicated sequence around position 65 (Fig. 2A). It is difficult to predict to what extent the insertions interfere with the overall protein fold. A general destabilisation would cause reduced nucleotide affinity and thus probably increased GTP loading. It is possible that the disturbances of the insertions are largely absorbed by the loop connecting β-sheet 3 and helix α2 (Fig. 2B). This loop constitutes a major component of the interaction surface with GEFs and thus strong impact on GEF interaction is expected (Fig. 2C). The insertions likely affect in particular the C-terminal part of the loop. The N-terminal part of the loop interacts with GAPs and contains Gln61, a residue involved in GTP hydrolysis (Fig. 2D). The insertions are not necessarily expected to be incompatible with GAP interaction, but impact on it is likely. The loop is spatially rather distant to the binding sites of RAS Association (RA) domains and RAS Binding Domains (RBDs) (Fig. 2E). These domains are found in RAS effectors, adapt ubiquitin folds, and interact with RAS•GTP in a similar manner^20^. The RasGEF SOS contains besides the catalytic site an allosteric site that binds to RAS•GTP (Fig. 2E). Binding of RAS to the allosteric site increases the catalytic activity of SOS resulting in a positive feedback^21,22^. The allosteric site in SOS is not constituted by an ubiquitin fold, but interacts with similar region as RAS effector proteins with an ubiquitin fold (Fig. 2E).

The clinical context indicated that VMOS RAS variants cause enhanced RAS signalling, but the outcome of the *in silico* analysis is not unambiguously supporting this expectation. In fact, it strongly suggested deficiencies in GEF catalysed nucleotide exchange, which would result in reduced signalling. An exact judgement of the functional consequences of the insertions thus requires experimental analysis. As the insertion observed in case 5 co-occured with p.Q61L, a classic pathogenic missense mutation, the following analysis is focused on the other VMOS RAS variants. The impact of the insertion on nucleotide exchange, effector binding, and GTP hydrolysis was systematically checked.

### Nucleotide exchange

**Figure 3:**
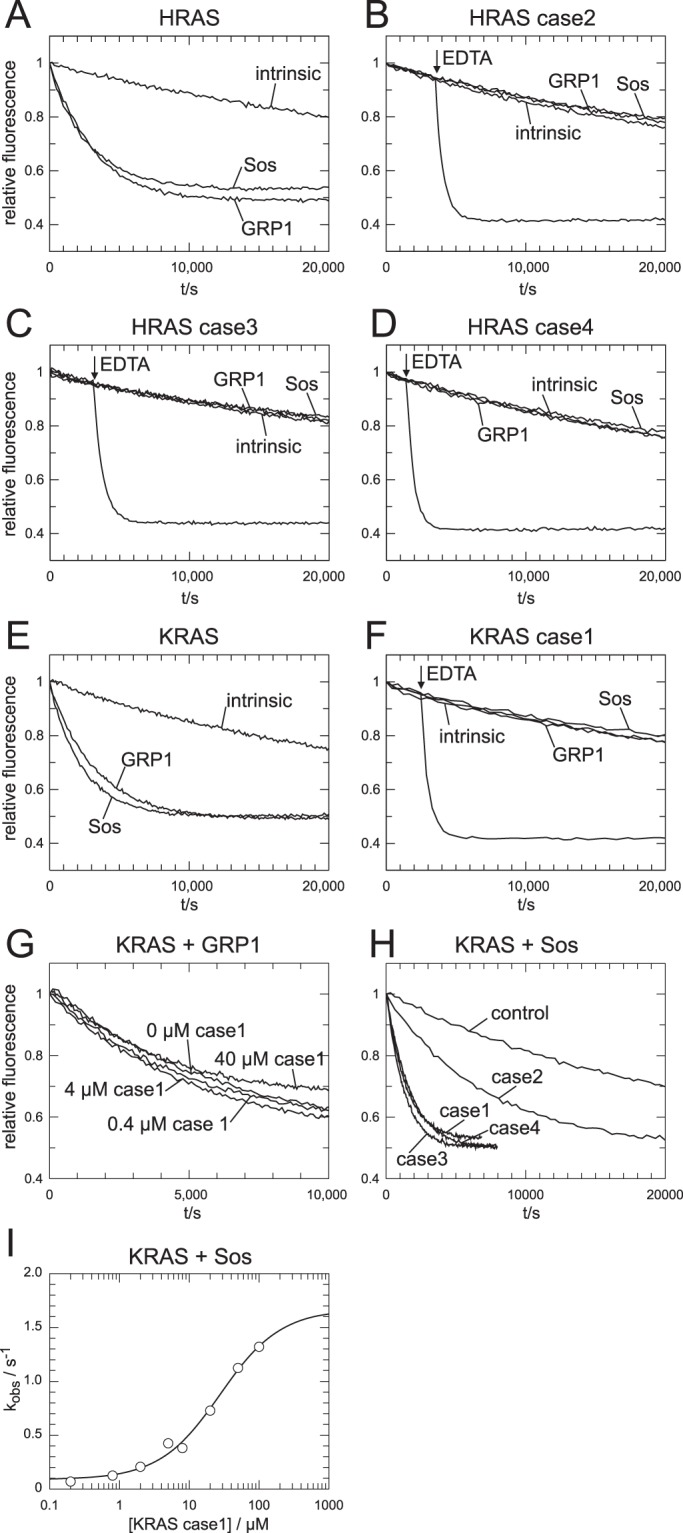
VMOS RAS variants are insensitive to GEFs. (**A**–**F**) Nucleotide exchange rates in the absence or presence of RasGRP1 (150 nM) or SOS (1.5 μM) of wild type (**A**,**E**) and variant RAS (**B**–**D**,**F**). For variant RAS an additional control was included to which EDTA was added at the indicated point in time. (**G**) RasGRP1 (150 nM) catalysed nucleotide exchange activity on wild type KRAS (200 nM) in the presence of various concentrations of the KRAS case1 variant. (**H**) SOS (150 nM) catalysed nucleotide exchange activity on wild type KRAS (200 nM) in the presence of variant RAS (20 μM) as indicated. Variant RAS was used as obtained from the protein purification and therefore in part loaded with GDP and in part with GTP. The portion of GTP loaded RAS was 64% (case 1), 71% (case 2), 39% (case 1), and 77% (case 4). *Note:* The reduced ability of the case 2 variant cannot be attributed to lower GTP loading. (**I**) SOS catalysed nucleotide exchange was measured as in (H) in the presence of various concentration of the case 1 KRAS variant. The decay of the fluorescence signal was fitted to a single exponential functions and the obtained rate constants (k_obs_) were plotted against the concentration of the KRAS variant.

To analyse nucleotide exchange, recombinant RAS was loaded with the fluorescent GDP analogue mGDP and nucleotide exchange was monitored in the presence of excess GDP as a decay of fluorescence intensity. The intrinsic dissociation rate from wild type RAS and VMOS RAS variants are indistinguishable (Fig. 3A–G). This suggested that the nucleotide affinity of the VMOS RAS variants is not altered. Unlike the classical codon 117 or 146 missense mutations, pathogenicity of the VMOS RAS variants does therefore not originate from increased GTP loading due to reduced nucleotide affinity and fast nucleotide exchange.

The GEFs SOS and RasGRP1 catalyse nucleotide exchange of wild type HRAS and KRAS (Fig. 3A,E). However, the exchange rates of the VMOS RAS variants remain unchanged in the presence of GEFs (Fig. 3B–D,F). To demonstrate proper nucleotide loading of the VMOS RAS variants, EDTA was added in a control reaction. One Mg^2+^ ion is bound to small G-proteins and co-ordinated in part by the negative charged oxygens from the phosphate moiety of the nucleotide. Upon complexation of Mg^2+^ by EDTA, the nucleotide affinity of the small G-proteins is drastically reduced, resulting in fast nucleotide dissociation. Indeed, all RAS variants were initially loaded with mGDP properly.

Even though SOS and RasGRP1 were unable to catalyse nucleotide exchange of the VMOS RAS variants, it is formally not excluded that a ternary complex between the GEF and the nucleotide loaded G-protein is formed. In such a scenario, the VMOS RAS variant could act as dominant negative by sequestering GEFs in an unproductive complex. To exclude this possibility, RasGRP1 mediated nucleotide exchange of KRAS•mGDP was measured in the presence of an up to 100 fold higher concentration of the case 1 KRAS variant (Fig. 3G). Indeed, the presence of the variant did not reduce the rate of RasGRP1 mediated nucleotide exchange of KRAS wild type.

When the same experimental set-up was used with SOS, a strong enhancement of SOS mediated nucleotide exchange of KRAS•mGDP was measured in the presence of KRAS and HRAS variants (Fig. 3H,I). This indicates that the variants were still able to activate SOS by binding to the allosteric site. It is for technical reasons not possible to compare the variants with wild type RAS, as wild type RAS would compete with KRAS•mGDP for the catalytic site. The case 2 HRAS variant seemed to be less potent in activating SOS if compared to the other variants, which could be explained by a reduced affinity for the allosteric site. In summary, as predicted by the *in silico* analysis, nucleotide exchange by GEFs was perturbed, but the variants were still able to activate SOS by binding to the allosteric site.

### Effector interaction

**Figure 4:**
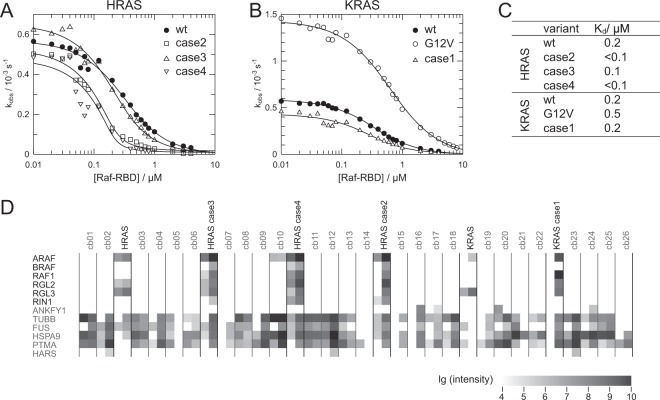
Analysis of effector interaction. (**A**,**B**) Nucleotide dissociation rates (k_obs_) of RAS loaded with the fluorescent GTP analogue mGppNHP were determined in the presence of various concentrations of Raf-RBD and plotted against the concentration of Raf-RBD. Solid lines, data fit. (**C**) Affinities of the interaction as determined from the data in (**A**,**B**). (**D**) GTPγS loaded G-proteins together with 26 different unrelated reference baits (cb01 to cb26) were immobilised and used for precipitation of interacting proteins from HeLa cells in duplicate. The obtained precipitations were analysed by mass spectroscopy. The summed peptide intensities of identified proteins were logarithmical transformed to the basis of 10 and are represented by shades of grey. *Note*: intensities smaller than 10^4^ are not obtained due to technical constrains. In total 1254 proteins were identified with more than 2 peptides. Here, only the identified known RAS effectors and a few “background” proteins are shown.

The interaction of G-protein and the nucleotide is stabilised in the ternary complex with the effector and in consequence the rate of nucleotide dissociation is reduced. This effect was used to analyse the interaction of wild type and VMOS RAS variants with the RAS Binding Domain of RAF (Raf-RBD) (Fig. 4A,B). RAS was loaded with the hydrolysis resistant fluorescent GTP analogue mGppNHp and the nucleotide dissociation rate was determined in the presence of excess GppNHp and various concentrations of Raf-RBD. Both HRAS and KRAS interacted with Raf-RBD with a K_d_ of 0.2 μM. The affinities for the interaction of case 1 and case 3 variants were very similar to the respective wildtype protein (Fig. 4A–C). The affinities of the case 2 and case 4 variants were increased, but as the titrations were performed at a concentration of 0.2 μM of the G-protein K_d_ values much lower than 0.2 μM could not be determined accurately. For comparison, KRAS p.G12V was included as a classical oncogenic variant. KRAS p.G12V interacted with a similar affinity with Raf-RBD as wild type KRAS (Fig. 4B,C). However, the intrinsic dissociation rate of mGppNHp was increased by a factor of about 3 compared to wild type and the VMOS RAS variants (Fig. 4B).

To confirm that the isolated RAS binding domain reflected the interaction of full length RAS effectors, wild type and VMOS RAS variants were loaded with the hydrolysis resistant GTP analogues GTPγS, immobilised, and used to precipitate proteins from HeLa cell extracts. The RAS effector proteins ARAF, BRAF, RAF1, RGL1, RGL2, RGL3 and RIN1 were identified in the precipitates obtained with wild type and all VMOS RAS variants but not with several unrelated control proteins (Fig. 4D), showing that at least in case of the identified proteins interaction with the full length effector was possible.

### GTP-hydrolysis

**Figure 5:**
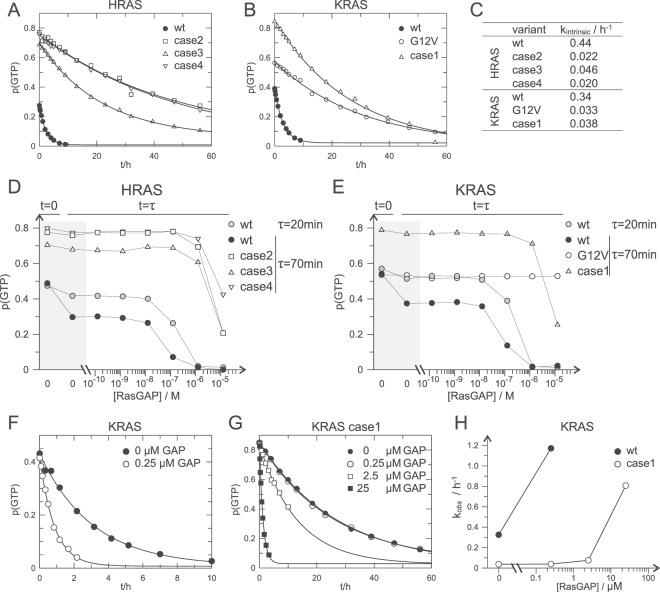
VMOS RAS variants displayed reduced GTPase activity. (**A**,**B**) The GTP fraction p(GTP) of the total nucleotide load of wild type and mutated HRAS (**A**) and KRAS (**B**) was followed over time. The time dependent decay of p(GTP) was fitted as single exponential decay (solid line) to determine the rate of intrinsic GTP hydrolysis k_intrinsic_. (**C**) Intrinsic GTP hydrolysis rates k_intrinsic_ as determined in (**A**,**B**). (**D,E**) RasGAP catalysed GTPase activity of wild type and variant HRAS (**D**) and KRAS (**E**). p(GTP) was determined at time zero and after τ = 70 minutes of incubation in the presence of various concentration of RasGAP. Wild type RAS was in addition analysed after τ = 20 minutes. Solid lines connect the data points belonging to the series of a RAS variant. (**F,G**) Time dependent GTP hydrolysis of KRAS wt (**F**) and KRAS (**G**) in the presence of various RasGAP concentration. The dependent decay of p(GTP) was fitted as single exponential decay (solid line) to obtain k_*obs*_. *Note*: Different scales are used in (**F**,**G**). (**H**) k_*obs*_ as obtained in (**F**,**G**) were plotted against the concentration of RasGAP. Solid lines connect the data point belonging to one series.

GTP hydrolysis was followed over time by terminating the reactions at different points in time, and GDP and GTP contents analysis by HPLC. The intrinsic GTP hydrolysis rates of VMOS RAS variants were reduced by a factor 10 relative to wild type (Fig. 5A–C). The same extent in reduction of the intrinsic hydrolysis rate was observed for the classical oncogenic KRAS p.G12V variant (Fig. 5B,C). The differences between wild type and mutated RAS were also reflected by the different GTP content at time zero, as GTP hydrolysis had already occurred during the procedure of nucleotide loading and further preparations of the proteins (Fig. 5A,B).

To analyse the effect of the VMOS RAS variants on GAP catalysed GTP hydrolysis, G-proteins were incubated in the presence of various concentration of RAS-GAP. GTP and GDP content was analysed after termination of the reaction after 70 minutes (Fig. 5D,E). In case of the wild type proteins, an additional series was recorded with termination after 20 minutes (Fig. 5D,E). The classical oncogenic variant KRAS p.G12V was taken along for comparison (Fig. 5E). RAS-GAP was still able to stimulate GTP hydrolysis of all analysed VMOS RAS variants, though stimulation seemed to be less efficient than with wild type HRAS and KRAS. The VMOS RAS variants were clearly distinct from KRAS p.G12V, as RAS-GAP was not able to stimulate GTP hydrolysis of KRAS p.G12V even at the highest concentration used (Fig. 5E).

Furthermore, KRAS wild type and the case 1 KRAS variant were subjected to a more detailed analysis for which GTP hydrolysis was monitored over time in the presence of various concentrations of RAS-GAP (Fig. 5E,G). While 0.25 μM of RAS-GAP enhanced the GTPase activity of KRAS wild type by a factor of about 3.5, no effect was observed on the case 1 variant. Enhancing effects with the case 1 variant were observed at 2.5 μM and 25 μM. At these concentrations GTP hydrolysis of KRAS wild type became too fast to be resolved. The dependency of GTP hydrolysis of the RAS-GAP concentration thus suggested that the affinity of the VMOS variants for GAPs was reduced.

### Functional characterisation of VMOS RAS variants

**Figure 6:**
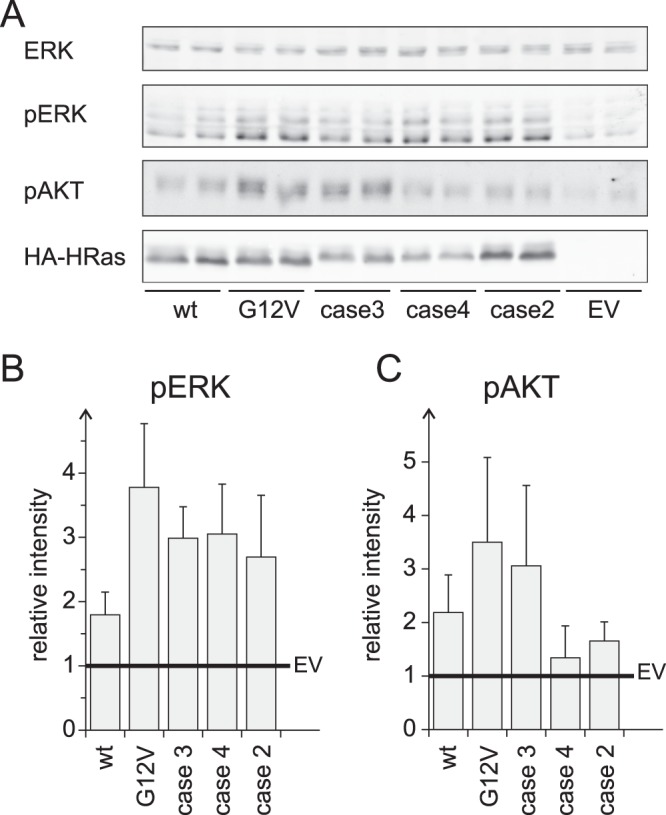
VMOS RAS variants enhance RAS signalling *in vivo*. (**A**) A14 cells were transfected with HA-tagged versions of wild type and mutated versions of HRAS as indicated. Cell lysates were subjected to SDS-page and Western blotting. Blots were probed with α-HA, α-ERK, α-pERK, and α-pAKT. Per experiment each transfection condition was duplicated (same transfection mix spilt of two dishes of cells). This figure shows one representative experiment and four independent experiments are shown in Supplementary Fig. 2. Full scans of the blots shown in this figure are provided as Supplementary Fig. 3. (**B**,**C**) Quantification of the levels of pERK (**B**) and pAKT (**C**) obtained in all five experiments. The intensity of the bands were integrated and background subtracted. Bar graphs with the intensity values obtained for the individual bands are shown in Supplementary Fig. 2. For each experiment the intensities of the duplicated transfections were averaged and normalised to the empty vector control resulting in five values per condition. Here, the average of these values and their standard deviation are shown as bar graphs.

The biophysical characterisation of the VMOS RAS variants showed two opposing effects. VMOS RAS variants appeared insensitive to the action of GEFs with a consequently decrease of signalling capability. On the other hand, VMOS RAS variants showed reduced intrinsic and GAP catalysed GTP hydrolysis with an increase of signalling capability as consequence. To analyse the net consequences of these opposing effects in the cellular context, A14 cells were transiently transfected with either HA-tagged versions of HRAS, HRAS p.G12V or VMOS variants of HRAS (Figs 6 and S1). Cell lysates were analysed for the level of phosphorylated ERK (pERK) to monitor RAS induced RAF activity. pERK levels were found to be low in empty vector controls and slightly increased in cells transfected with wild type HRAS. HRAS p.G12V and the VMOS RAS variants caused a clear induction of pERK levels (Fig. 6A,B and Supplementary Fig. 2). Furthermore, cell lysates were analysed for phosphorylated AKT (pAKT) at Ser473 to monitor RAS induced PI3K activity. Whereas HRAS p.G12V and the case 3 variant induced phosphorylation of AKT no enhancement was observed with the case 2 and case 4 variant (Fig. 6A,C).

### Meta-analysis of comparable in-frame insertions in RAS genes

**Figure 7:**
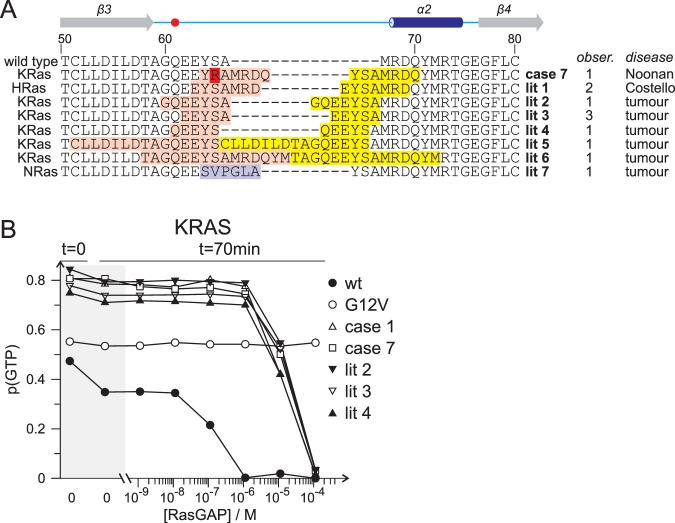
GAP catalysed GTPase activity of VMOS related RAS variants. (**A**) Sequence alignment as in Fig. 2A of a KRAS variant found in a Noonan patient (case 7; this study), a HRAS variant found in two patients with Costello (lit 1;^23,24^) and of 6 variants of either KRAS or NRAS found in 8 different tumour samples (lit 2–7;^25–30^). The number of independent observations of the variant and the context is indicated on the right of the alignment. Colour coding as in Fig. 2A. (**B**) p(GTP) was determined at time zero and after 70 minutes of incubation in the presence of various concentration of RasGAP as in Fig. 5.

An in-house database search for insertions comparable to the VMOS RAS variants revealed one *in-frame* insertion in *KRAS* in a case suspected for Noonan syndrome (Fig. 7A). Furthermore, a screen of the current literature and public databases COSMIC and ClinVar identified a total of 7 different insertions in 10 cases (Fig. 7A). One insertion is reported for two independent cases of Costello syndrome^23,24^ and 6 different insertions are reported for 8 tumour samples^25–30^. Though these variants are similar to the VMOS RAS variants, it is possible that they have acquired a stronger signalling capability. For example, it seems that at least some of the insertions found in tumours extended more to the N-terminal side compared to the VMOS RAS variants. In consequence, the impact of these insertions on the catalytic Gln61 might be stronger. To test this, RasGAP catalysed GTP hydrolysis was monitored with the KRAS variant found in Noonan and three selected variants described in tumours (Fig. 7B). All four variants displayed a very similar behaviour as VMOS RAS variants, excluding the possibility that the variants found in tumours enhance signalling stronger than the VMOS RAS variants.

## Discussion

From a cohort of 299 samples of VMOS, 15 cases with variants in either *HRAS*, *KRAS* or *NRAS* were identified. While in nine of these a classical or semi-classical pathogenic missense variant was present, six cases harboured an insertion of 7 to 10 amino acid residues around position 65 (Fig. 1). Thus classic pathogenic variants and this type of insertion variants were found at a ratio of 2:1. An extensive literature search and a search of the COSMIC database identified in total ten reported cases with similar insertions in either *HRAS, KRAS* or *NRAS* (Fig. 7A). One insertion was found in two independently reported patients with Costello syndrome^23,24^. The remaining eight reported variants including in total six different insertions were detected in tumour samples. The low number of reported similar insertions is surprising as *RAS* genes are extensively sequenced in tumour specimens. In part, this might be explained by the difficulties in alignment and variant detection of such insertions in NGS-based analysis and the focus on hotspot locations in the *RAS* genes that do not extend towards the positions around amino acid 65. However, also in our routine diagnostics for oncology requests, no such insertion variants were identified (Fig. 1). Therefore it is possible that the relative frequencies of *RAS* variants detected in different diseases reflect the extent of mutational activation.

The biophysical characterisation performed here suggested that these type of insertion variants are weakly but constitutively activating. The GEFs SOS and RasGRP1 did not display any activity to the variants. It is expected that this holds true for other known RasGEFs as well, since these GEFs are structurally related^31^. The crystal structure of the RAS•SOS complex demonstrates that the region between residue 60 and 70 of RAS is involved in extensive interactions with SOS^32^, strongly suggesting that insertions in this region are not tolerated by GEFs. Furthermore, point mutations in this region impact on the selectivity of the interaction between GEF and G-protein^33^. The RAS *in-frame* insertion variants thus seemed to be decoupled from activating cellular signals via the regulation of GEFs. Opposite to RAS proteins mutated at position 12 or 13, the RAS *in-frame* insertion variants were solely dependent on intrinsic nucleotide exchange that did not differ from wild type. Any increase in the GTP loaded fraction of the RAS *in-frame* insertion variants therefore has to be a consequence of impaired GTP hydrolysis. The intrinsic GTPase activity of the insertion variants was reduced by a factor 10. This is comparable to the p.G12V variant. RasGAP catalysed GTP hydrolysis was impaired but not abolished in the RAS *in-frame* insertion variants, whereas this was the case for p.G12V variants. Overall the RAS *in-frame* insertion variants are less likely to undergo transition from the GDP bound state to the GTP bound state than wild type RAS or RAS mutated at position 12 or 13. Transition from the GTP bound state to the GDP bound state occurs less likely in RAS *in-frame* insertion variants than in wild type but more likely in RAS mutated at position 12 and 13. The experiments in A14 cells would be in agreement with a shift of the net balance between the GDP and the GTP bound state to the GTP bound state in the RAS *in-frame* insertion variants.

Overall, VMOS variants seemed able to interact with effector proteins, though the interaction may be attenuated to different degrees for the individual VMOS variants. The case 2 and case 4 variants displayed increased affinity for the isolated Ras Binding Domain of RAF (Fig. 4A–C). On the other hand, the case 2 variant seemed to be less efficient in enhancing SOS activity by binding to the allosteric site of SOS (Fig. 3H) and the case 2 and the case 4 variants did result in phosphorylation of ERK but not AKT if transfected in A14 cells (Fig. 6). The divergence of these effects may in part be explained by the steric demand of the insertions. Based on the available structural information it is expected that the insertions do not perturb the core effector interaction surface in RAS. However, clashes may occur with effector-specific parts in close proximity to the binding site (Fig. 2E).

The clinical manifestation of RAS variants is influenced by additional parameters. The type of the affected cell and the origin of the variant, germline or somatic, are obvious important factors. Of speculative nature is the influence of biochemical different behaviours of variants. For example, cells might react differently to the RAS *in-frame* insertion variants with a constitutive basal increase of RAS•GTP levels than to p.G12 variants where in addition RAS•GTP levels overshoot in response to GEF activation.

The strongly activating effect of RAS proteins mutated at positon 12 or 13 typically results in enhanced cell growth and drives the formation of tumours. These variants do not commonly occur in the germline and if so, phenotypes are severe. From six published RAS *in-frame* insertion variants in tumours literature variants #2, #3 and #4 were tested here for GTPase activity (Fig. 7A). All three tested variants displayed very similar deficiencies regarding GTP hydrolysis as VMOS RAS variants (Fig. 7B). In agreement with this, enhanced RAS signalling was reported in cell lines upon expression of the literature variants #1, #2, and #3^23,25^. It is not proven, that these variants indeed act as drivers of tumorigenesis. However, it seems most plausible that the risk of tumour formation increases with the extent to which RAS signalling is enhanced. But even strongly enhancing variants do not automatically cause cancer. This was reflected by the relative low number of classical RAS variants in the cohort of 299 VMOS cases, compared to cases of tumour diagnostics. On the other hand, weakly enhancing RAS variants of the VMOS type only rarely manifest themselves in tumours and are thus found with much higher frequencies in clinical manifestations of mild enhanced tissue growth.

In conclusion, we have characterised a novel type of insertions in *RAS* genes that weakly enhance constitutive signalling. All analysed variants were unresponsive to GEFs and displayed reduced intrinsic and GAP catalysed GTPase activity. These variants were likely causative in about 2% of the patients with atypical VMOS analysed here. It remains speculative whether and with which frequency these variants also occur in more common cases of VMOS, which usually do not require tissue examination and are thus not subjected to NGS as standard. However, newly identified pathogenic variants in atypical VMOS are of particular clinical interest, as diagnosis and treatment of atypical cases may be very challenging. Similar variants are found in incidental cases of RASopathies. Thus, this work adds a new class of RAS variants to the clinical spectrum that should be included in diagnostic sequencing.

## Material and Methods

### Patient cohort and next generation sequencing

In the period from January 2016 to June 2018, a total of 299 samples with clinical, histological or radiological evidence of VMOS were successfully screened. Study protocols on the clinical, histological and molecular data was performed with approval of the medical ethical board of the Radboudumc, Nijmegen, the Netherlands (number 2016–2310). Human tissue samples were obtained with oral informed consent for routine diagnostic analysis, which includes sequence analysis of affected tissue to detect acquired mutations. The need of written consent was waved by the ethical board as incidental findings were not expected. The identified acquired RAS variants were subject to further investigation through *in vitro* experiments using unrelated cell lines. No human tissue samples were used for experimental purposes. DNA isolation and library preparation were performed as previously described^34^. Libraries for sequencing on the NextSeq 500 (Illumina) were generated using Single Molecule Molecular Inversion Probes (smMIPs). This method uses unique molecule identifiers to allow consensus based error correction and the deduction of the actual number of sequenced gDNA molecules. This allows both sensitive detection of variants down to 1% variant allele frequency and specification of the sensitivity of sequencing on a case by case basis For the *RAS* genes, the analysis focused on hotspots and surrounding sequences of *HRAS* (NM_005343.2): codon 12, 13, 59 and 61, *KRAS* (NM_004985.4): codon 12, 13, 59, 61, 117 and 146 and *NRAS* (NM_002524.4): codon 12, 13, 59, 61, 117 and 146.

For 224 cases, the panel consisted of a cancer hotspot panel targeting the following hotspots and surrounding sequences in the following genes: *AKT1, BRAF, CTNNB1, EGFR, ERBB2, GNA11, GNAQ, GNAS, H3F3A, H3F3B, HRAS, IDH1, IDH2, JAK2, KIT, KRAS, MLP, MYD, NRAS, PIK3CA, PDGFRA*. For 75 cases, analysis was performed with a dedicated VMOS panel targeting hotspots and surrounding sequencing in the genes *AKT1, AKT2, ATK3, BRAF, GNA11, GNA14, GNAQ, GNAS, HRAS, IDH1 IDH2, KRAS, MTOR, NRAS, PIK3CA, PIK3R2* and *TEK* and >90% of coding and splice sequences of *PTEN* and *RASA1*.

### Purification of recombinant protein

HRAS (aa 1–166, *Homo sapiens*) was expressed from the ptac plasmid in the bacterial strain CK600K and purified as described^35^. KRAS (aa 1–170, *Homo sapiens*) was expressed as GST-fusion proteins from pGEX6P3 and the GST-tag was removed by cleavage with PreScission Protease as essentially as described^36^. HRAS and KRAS mutants were generated by QuikChange mutagenesis and purified as the wild type proteins. SOS1 (aa 564–1049, *Homo sapiens*) and RasGRP1 (aa 1–461, *Rattus norvegicus*) were expressed as HIS-tagged proteins using the pET vector system as described^33^. RasGAP (aa 722–1056, *Homo sapiens*) were expressed as GST-tagged proteins from the pGEX6P3. Protein production in CK600K bacteria was induced by 100 nM IPTG (isopropyl β-D-1-thiogalactopyranoside) at 25 °C. After 20 hrs, the bacteria were collected by centrifugation, resuspended in buffer A (50 mM TrisHCl pH 7.5, 50 mM NaCl, 5% glycerol and 5 mM β-mercaptoethanol) supplemented with 100 μM PMSF and lysed by sonication. The lysate was cleared by centrifugation and applied to glutathione agarose column equilibrated in buffer A. The column was washed with 10 to 15 volumes of buffer B (50 mM TrisHCl pH 7.5, 400 mM NaCl, 5% glycerol and 5 mM β-mercaptoethanol), 5 volumes of buffer A and eluted with 20 mM glutathione in buffer A. The protein containing fractions were pooled and concentrated. Cleavage of the GST tag was performed by the addition of PreScission Protease was added with a ration 1:500 (g protease/g protein) at 4 °C overnight. RasGAP was further purified by gel-filtration on a Superdex 75 26/60 column (GE-Healthcare) equilibrated with buffer C (50 mM TrisHCl pH 7.5, 400 mM NaCl, and 5 mM β-mercaptoethanol). RasGAP containing fractions were polled concentrated and remaining GST was removed by passing the protein over a glutathione agarose column.

### GEF mediated nucleotide exchange

GEF mediated nucleotide exchange was determined as described previously^33^. Briefly, reaction were performed with 200 nM of G-protein•mGDP in the presence of 20 μM GDP in buffer containing 50 mM TrisHCl pH 7.5, 100 mM NaCl, 5 mM MgCl_2_, 5% glycerol, and 5 mM DTT at 20 °C. RasGRP1 was used at 150 nM, Sos at 1500 nM or 150 nM, and EDTA at 10 mM.

### G-protein effector interaction

G-proteins were loaded with the hydrolysis resistant fluorescent GTP analogue mGppNHp^33^. 200 nM G-protein•mGppNHp were incubated in the presence of 20 μM GppNHp and various concentrations of the RBD of Raf at 35 °C in buffer containing 50 mM Tris HCl, ph 7.5, 50 mM NaCl, 5 mM MgCl_2_, 5 mM DTT and 5% glycerol. Fluorescence intensity was monitored over time and the fluorescence traces fitted as single exponential decay with offset to obtain the rate constant k_obs_. k_obs_ were plotted against the concentration of Raf-RBD and affinities were determined by data fitting as described^35^.

### Mass spectroscopy

After clean up peptides obtained from the trypsin digested precipitates were separated on a 30-cm pico-tip column (50 μm ID, New Objective) packed with 3 μm aquapur gold C-18 material (Dr. Maisch) by applying a gradient (7–80% ACN 0.1% FA, 140 min), delivered by an easy-nLC 1000 system (LC120, Thermo Scientific), and electro-sprayed directly into an LTQ Orbitrap Mass Spectrometer (Velos, Thermo Scientific). Raw files were analysed with the MaxQuant software version 1.5.1.0. with oxidation of methionine set as variable and carbamidomethylation of cysteine as fixed modification. The Human protein database of UniProt was searched with peptide and protein false discovery rate set to 1%.

### GTPase activity

G-proteins were loaded with GTP as described^33^. G-proteins were incubated at a concentration of 250 μM in buffer containing 50 mM TriHCl, pH 7.5, 50 mM NaCl, 5 mM MgCl_2_, 5 mM β-mercaptoethanol and 2.5% glycerol at 25 °C in 5 μl aliquots. The reaction was terminated by the addition of 100 μl acetonitrile at various points in time and immediately flash frozen in liquid nitrogen. The samples were concentrated to dryness in at reduced pressure and re-suspended in 100 μl water. To remove protein 10 μl of a suspension containing 50% OligoR3 C18 material in methanol were added. After 20 minutes incubation samples were filtered through a fritted 96 well plate (Nalgene), concentrated to dryness under reduced pressure and re-suspended in 30 μl water. 20 μl of sample were injected for analysis by HPLC (Ultimate 3000, Thermo Scientific) and nucleotides separated on a C18_A0.3 μm 150 × 4.6 mm column (Dr. Maisch) with 0.5 ml/min isocratic flow of buffer containing 100 mM KPO_4_ pH 6.5, 10 mM Terabutylammoniumbromide and 10% acetonitrile and absorbance was detected at a wavelength of 260 nm. The identity of peaks were confirm by GDP and GTP standards. Peaks were integrated and p(GTP) was determined as peak integral GTP/(peak integral GTP + peak integral GDP).

### ERK-activation and Western blotting

A14 cells^37^ were cultured in 6 polystyrene well plates (Corning) in DMEM high glucose medium (Lonza) supplemented with 10% fetal bovine serum (Bodinco BV), 100 U/l penicillin streptomycin mixture (Lonza), and 20 mM L-glutamine. Cells were transfected with pMT2-HA vector or vector containing the indicated RAS variants with extreme gene (Roche) following the manufactures instructions. Two days after transfection and growth in full medium cells were harvested by scraping into 1X SDS sample puffer. Cell lysates were subjected to SDS page and blotted to PVDF membrane. Expression of RAS variants was monitored via the HA-tagged with a homemade monoclonal mouse anti-HA antibody (12CA3). Phosphorylated ERK was monitored with monoclonal rabbit antibody P-p44/42 MAPK (Cell Signalling 4370), total ERK with a homemade polyclonal rabbit antibody (6D3), and phosphorylated AKT with the monoclonal rabbit antibody pAKT S473 D9E (Cell Signalling 4060). For detection fluorescently labelled secondary anti mouse or anti rabbi antibodies were used (Alexa Fluor 680 and Alexa Fluor 800, Thermo Fisher Scientific GE). Fluorescence was read with a Typhoon scanner (Amersham GE) with a pixel size of 10 μm × 10 μm (corresponding to 2540 dpi) and a detection range of 16 bit. In figures the information containing range is visualised in an 8 bit gradient of grey scale. None orthogonal transformations and scaling in figure preparation was kept to a minimum.

### Ethical statement

The Study was performed with approval of the medical ethical board of the Radboudumc, Nijmegen, the Netherlands (number 2016–2310) and was in accordance with the Code of Conduct of the Federation of Medical Scientific Societies in the Netherlands. Oral informed consent was obtained from all patients.

## Supplementary information

supplementary information
