# Supplementary material for: Functional characterisation of a novel class of in-frame insertion variants of KRAS and HRAS
Source: Sci Rep. 2019 Jun 3;9:8239. doi: 10.1038/s41598-019-44584-7 (PMC6547725; doi:10.1038/s41598-019-44584-7)
Supplement: Supplementary file 1 — supplementary information [file 41598_2019_44584_MOESM1_ESM.pdf]

Supplementary Information for

Functional characterisation of a novel class of in-frame insertion variants of KRAS and HRAS

Astrid Eijkelenboom, Frederik M.A. van Schaik, Robert M. van Es,
Roel W. Ten Broek, Tuula Rinne, Carine van der Vleuten, Uta Flucke,
Marjolijn J. L. Ligtenberg, and Holger Rehmann

Case report of patients with insertions in *RAS* genes

Case 1

KRAS: c.187_207dup (p.(Glu63_Asp69dup)); VAF: 4%

55 years old female with a congenital erythematous macula of the right cheek/ maxilla with progressive livid swelling.

US: limited visibility; vascular malformation not excluded.

MRI: vascular malformation not excluded; indication for a small AVM.

Histological findings: Skin tissue with an increased amount of small to medium sized veins. Classified as veno-lymphatic malformation.

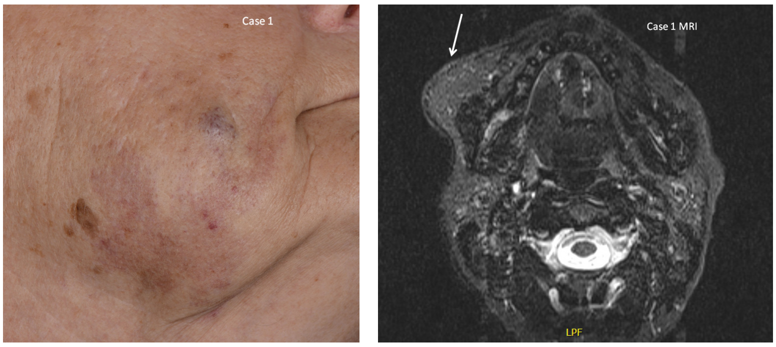

(*Left*) Clinical image of right cheek/ maxilla: erythematous macula with livid swelling. (*Right*) MRI: Fat-suppressed T2, Transversal image of the head-neck region: arrow points to deviating contour of the right cheek, indicating tissue swelling, mainly caused by the subcutaneous tissue abnormality. No clear flow voids. No certain vascular malformation. Probably a small arterio-venous malformation.

Case 2

HRAS: c.198_224dup (p.(Met67_Gly75dup)); VAF: 4%

24 years old male with a congenital livid macula at the left ankle. Progression of swelling during puberty to a plaque-like tumorous lesion with a lumpy aspect and a size of 5.5 x 8.5 cm.

US: vascular malformation not excluded. Indications for venous or mixed picture, not typical for AVM or lymphatic malformation.

MRI: capillary malformation with probable minimal flow based on the dilated venous structures are observed. No evidence for a tumour process.

Histological findings: Dermal and subcutaneous tissue with multiple partly lobularly arranged small atypical, both arterial and venous, vessels. Classified as vascular malformation, GLUT-1 negative.

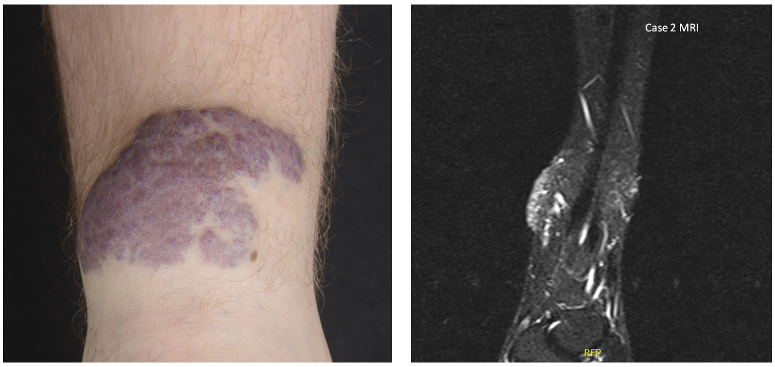

(*Left*) Clinical image of livid swelling of the left ankle with a lumpy aspect. (*Right*) MRI: Fat-suppressed T2, coronal image of the lower leg: on the ventral side, skin thickening of a few millimetres. Below dilated venous structures. No flow voids. The image fits best with a capillary malformation with probably minimal flow based on the dilated venous structures. No indications for a tumour process.

Case 3

HRAS: c.208_209insGGTGGGAGTACAGCGCCATGCGGGACC (p.(Asp69_Gln70ins9)); VAF: 4%

50 years old male with a painful and growing swelling of the buttocks with a size of 10 x 10 cm; initially considered as lipoma but vascular anomaly was diagnosed after resection.

MRI: very diffuse vascular anomaly, with a lot of flow-voids. But no AVM at angiography; more like a VM. Treatment as if it was a VM (with embolisation) did not have effect; progression of growth.

Histological findings: Subcutaneous fat tissue wherein multiple thin walled vessels, mainly venules and capillaries. Classified as vascular malformation.

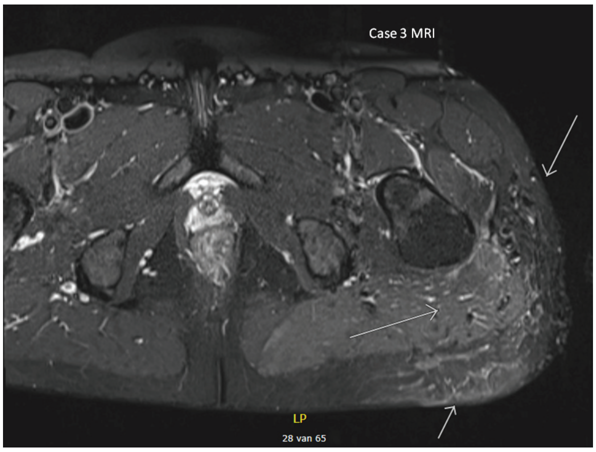

MRI T2, transverse image of the trunk/ pelvis: arrow points to a diffuse vascular anomaly, with many flow-voids.

Case 4

HRAS: c.215_216insCTCCAGCGCCATGCGGGACCAGTACAT (p.(Tyr71_Met72ins9)); VAF: 10%

26 years old male. Lesion incidentally found during US: a heterogeneous mass present at the abdominal wall of at least 9 x 4.2 x 4.8 cm, unsharply demarcated, with central flow, suitable for vascular anomaly.

MRI: Intramuscular tumour starting from the rectus abdominis right. Differential diagnosis is thought to be a myxoma, desmoïd or still sarcoma.

Histological findings: Soft tissue wherein a vascular lesion composed of both smaller and larger vessels and capillary convolutes. Classified as intramuscular capillary malformation, GLUT-1 negative.

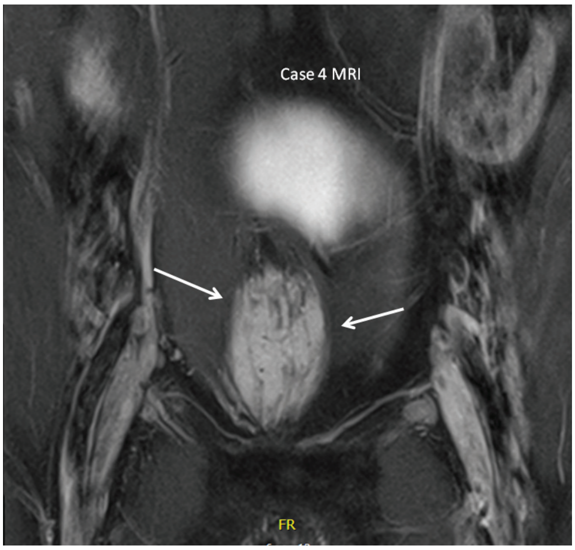

MRI T2, coronal image of trunk/ abdomen: arrow points to intramuscular vascular mass in the right muscular rectus abdominis.

Case 5

HRAS: c.[182A>T;187_216dup] (p.[(Gln61Leu; Glu63_Met72dup)]); VAF: 13%

17 years old male. Congenital, progressive and painful skin coloured, slightly bluish, soft-tissue swelling of the lower arm. Not typically a VM at palpation. Treatment as if it was a VM (with embolisation) did not have effect.

US: intramuscular, very vascular swelling

MRI: venous malformation in the musculus flexor carpi ulnaris with a size of 5.4 x 3.2 x 1.3 cm.

Histological findings: Skeletal muscle wherein a heterogeneous vascular lesion composed of arteries, veins, venules and capillaries. Classified as vascular malformation.

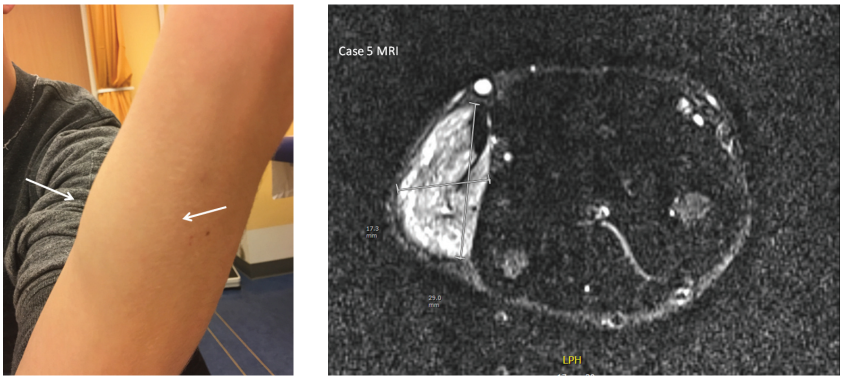

(*Left*) Clinical image of a skin coloured, slightly bluish, soft-tissue swelling of the lower arm. (*Right*) MRI T2 with fat suppression, transverse image of the left arm: large subcutaneous lesion on the side of the forearm with dimensions of 3 by 17 mm, over a distance of almost 6 cm: venous malformation in de musculus flexor carpi ulnaris.

Case 6

HRAS: c.208_209insGGTGGTACAGCGCCATGCGGGACC (p.(Asp69_Gln70ins8)); VAF: 7%

55 years old male. Since more than 20 years a progressive painful stabbing swelling at the flank characterised as congenital and low-flow malformation.

US: unsharply demarcated echo-dense configuration between the transverse abdominal muscles suspect for an inter / intramuscular lipoma. Size: 8 x 7 cm * 17 mm

MRI: intramuscular hemangioma

Histological findings: Subcutaneous and intramuscular tissue wherein a vascular lesion consisting of larger and smaller veins, capillaries and a lymphatic component. Classified as vascular malformation.

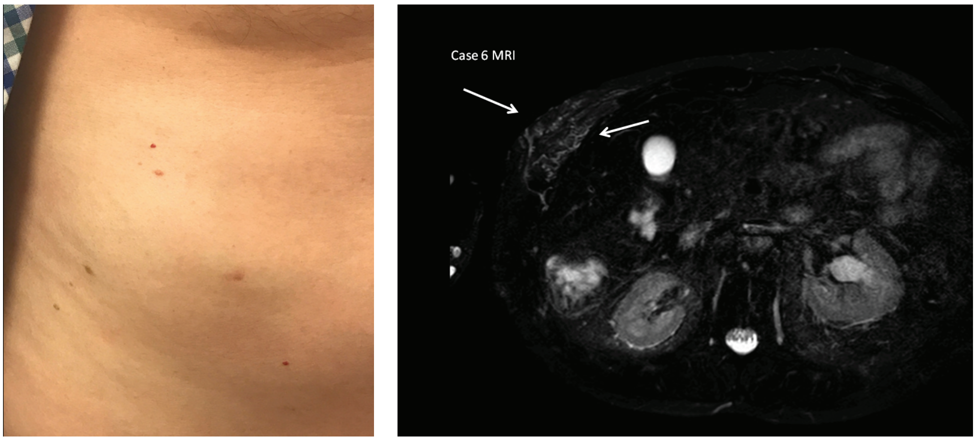

(*Left*) clinical image of swelling of about 7 cm at the flank. (*Right*) MRI Transverse image of the trunk/ abdomen: arrow points to diffuse subcutaneous and partly intramuscular vascular mass; intramuscular vascular anomaly.

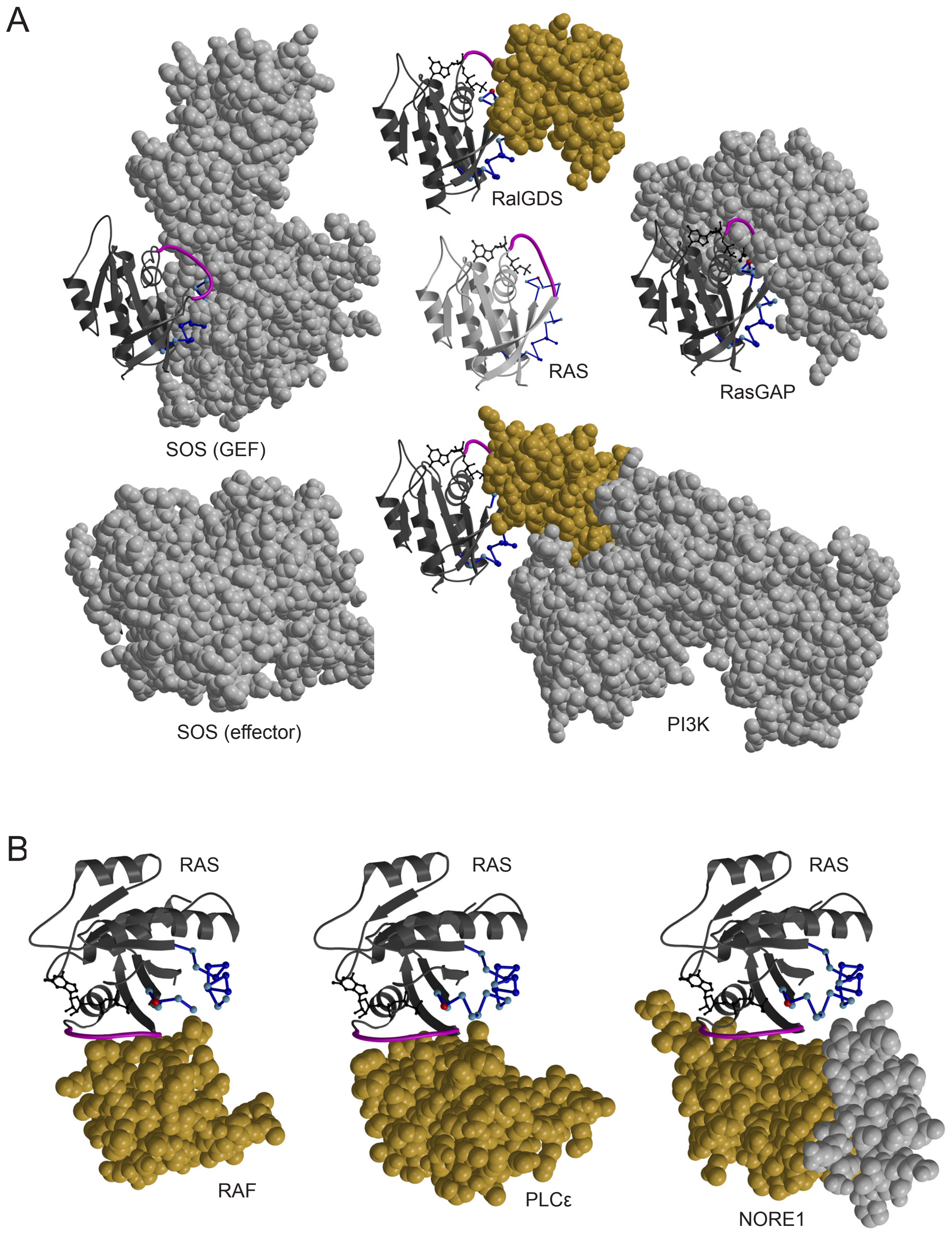

Supplementary Figure 1 | (**A**) Same structures as shown in Fig. 2B-E, but with Ras in the same orientation in all cases and without trimming. *Note*: The Ras molecule is masked up with SOS if bound to the allosteric site of SOS (panel SOS (effector)). (**B**) Extension of Fig. 2E. Structure of Ras in complex with the ubiquitin folds of RAF (left, pdb 4g0n), PLCε (middle, pdb 2c5l), and Nore1 (right, pdb 3ddc).

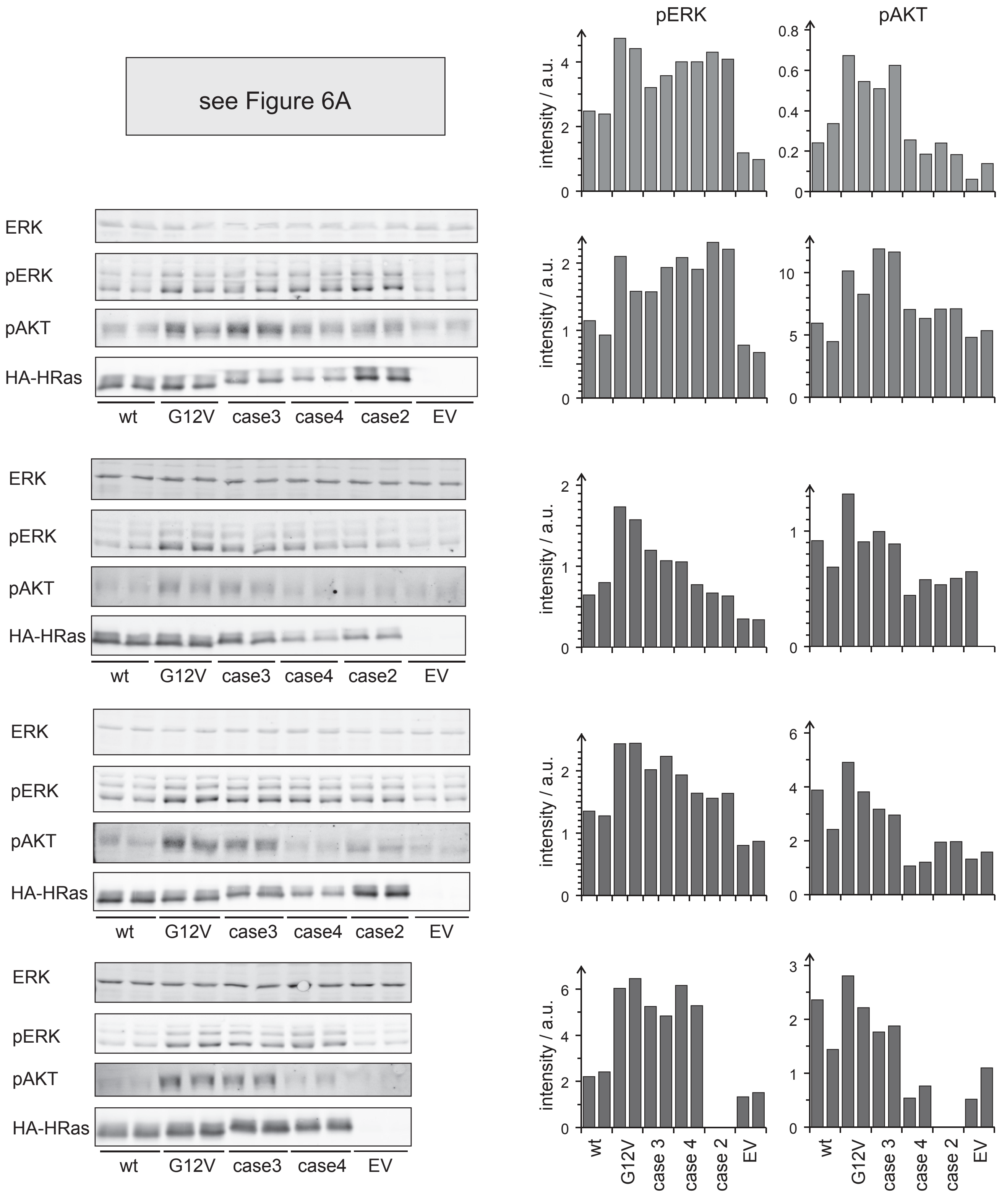

Supplementary Figure 2 | Independent replications of the experiment presented as Figure 6 and quantification of the experiments. The intensity of the bands were integrated and background subtracted. Bar graphs of the background subtracted intensities of each individual band are shown right to the blots.

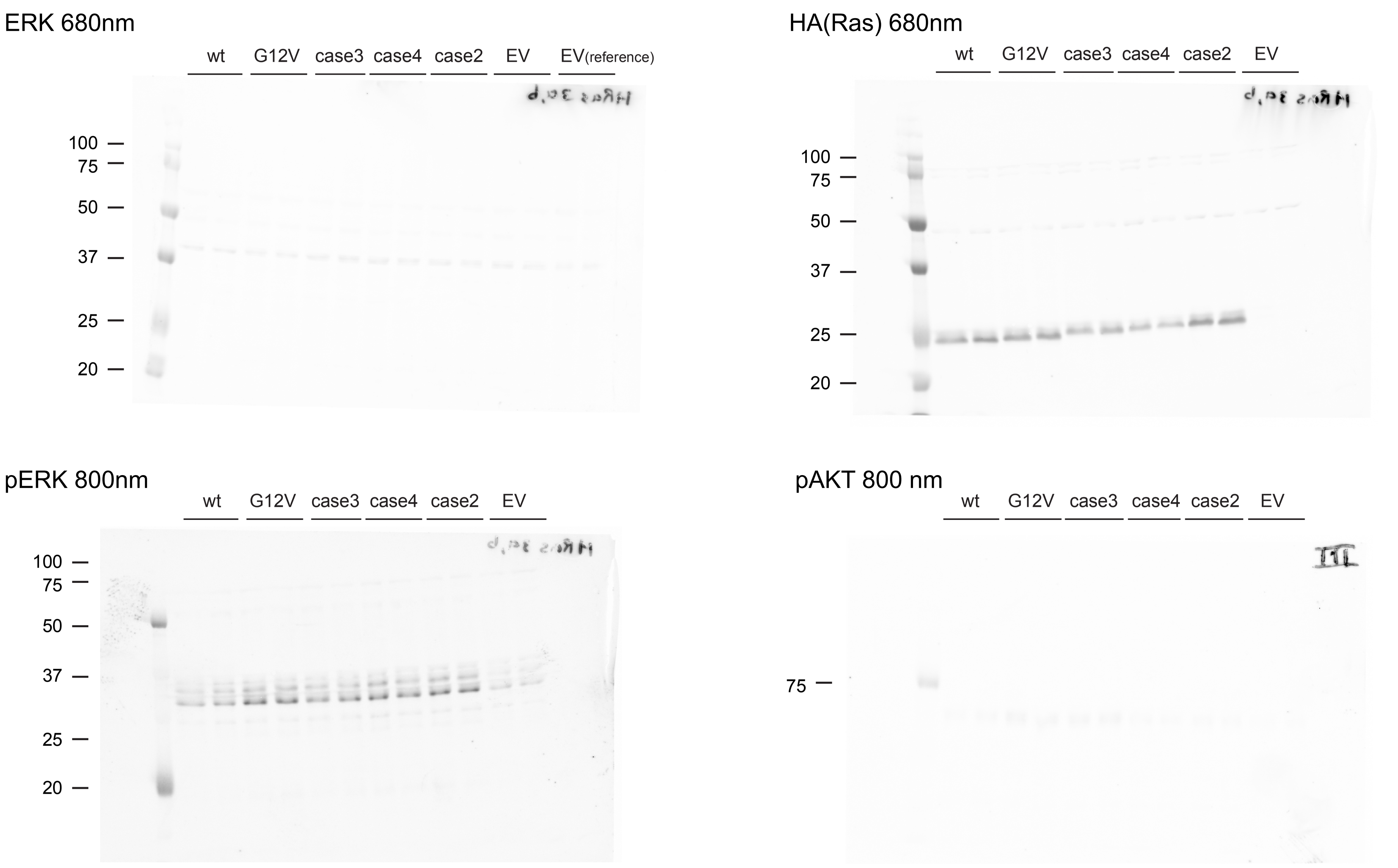

Supplementary Figure 3 | Scan of the full PVDF membranes stained with fluorescently labelled secondary antibodies from the blots presented in Figure 6. The original resolution of 1000 dots per cm was reduced to 200 dots per cm to obtained source files for the conversion into pdf. Scans are presented without any transformations. The full detection range of 16 bit is represented in an 8 bit gradient of grey scale. Note, the appearance of the marker bands (Precision Plus Protein Dual Color Standards, BioRad) depends on the detected wavelength indicated in the figure.
